# Distinct negative-sense RNA viruses induce a common set of transcripts encoding proteins forming an extensive network

**DOI:** 10.1128/jvi.00935-24

**Published:** 2024-09-16

**Authors:** Nina Hofmann, Marek Bartkuhn, Stephan Becker, Nadine Biedenkopf, Eva Böttcher-Friebertshäuser, Karina Brinkrolf, Erik Dietzel, Sarah Katharina Fehling, Alexander Goesmann, Miriam Ruth Heindl, Simone Hoffmann, Nadja Karl, Andrea Maisner, Ahmed Mostafa, Laura Kornecki, Helena Müller-Kräuter, Christin Müller-Ruttloff, Andrea Nist, Stephan Pleschka, Lucie Sauerhering, Thorsten Stiewe, Thomas Strecker, Jochen Wilhelm, Jennifer D. Wuerth, John Ziebuhr, Friedemann Weber, M. Lienhard Schmitz

**Affiliations:** 1 Bioinformatics and Systems Biology, Justus Liebig University Giessen, Giessen, Germany; 2 Biomedical Informatics and Systems Medicine Science Unit for Basic and Clinical Medicine, Justus Liebig University Giessen, Giessen, Germany; 3 Institute for Lung Health (ILH), Justus Liebig University Giessen, Giessen, Germany; 4 Institute of Virology, Philipps-University Marburg, Hans-Meerwein-Straße, Marburg, Germany; 5 Institute for Virology, FB10-Veterinary Medicine, Justus Liebig University Giessen, Giessen, Germany; 6 Institute of Medical Virology, FB11-Medicine, Justus Liebig University Giessen, Giessen, Germany; 7 Genomics Core Facility, Institute of Molecular Oncology, Philipps University of Marburg, Marburg, Germany; 8 Institute of Molecular Oncology, Philipps University of Marburg, Marburg, Germany; 9 Institute of Biochemistry, FB11-Medicine, Justus Liebig University Giessen, Giessen, Germany; University Medical Center Freiburg, Freiburg, Germany

**Keywords:** NSV, host cell response, transcription, protein network, RNA-seq

## Abstract

The large group of negative-strand RNA viruses (NSVs) comprises many important pathogens. To identify conserved patterns in host responses, we systematically compared changes in the cellular RNA levels after infection of human hepatoma cells with nine different NSVs of different virulence degrees. RNA sequencing experiments indicated that the amount of viral RNA in host cells correlates with the number of differentially expressed host cell transcripts. Time-resolved differential gene expression analysis revealed a common set of 178 RNAs that are regulated by all NSVs analyzed. A newly developed open access web application allows downloads and visualizations of all gene expression comparisons for individual viruses over time or between several viruses. Most of the genes included in the core set of commonly differentially expressed genes (DEGs) encode proteins that serve as membrane receptors, signaling proteins and regulators of transcription. They mainly function in signal transduction and control immunity, metabolism, and cell survival. One hundred sixty-five of the DEGs encode host proteins from which 47 have already been linked to the regulation of viral infections in previous studies and 89 proteins form a complex interaction network that may function as a core hub to control NSV infections.

**IMPORTANCE:**

The infection of cells with negative-strand RNA viruses leads to the differential expression of many host cell RNAs. The differential spectrum of virus-regulated RNAs reflects a large variety of events including anti-viral responses, cell remodeling, and cell damage. Here, these virus-specific differences and similarities in the regulated RNAs were measured in a highly standardized model. A newly developed app allows interested scientists a wide range of comparisons and visualizations.

## INTRODUCTION

Negative-strand RNA viruses (NSVs) are responsible for a substantial proportion of human infectious diseases and often have a zoonotic origin (1–3). Due to their comparatively high mutation rate, they are genetically variable and can rapidly adapt to environmental changes (4, 5). Many medically important viral pathogens are members of the NSV families *Orthomyxoviridae*, *Paramyxoviridae*, *Filoviridae*, *Arenaviridae*, or *Phenuiviridae* (the latter two belonging to the newly established order *Bunyavirales*) (https://talk.ictvonline.org/taxonomy/).

Mammalian cells sense infecting RNA viruses by recognizing their surface structures or the viral RNAs using a broad spectrum of pattern recognition receptors (PRRs) located at the cell membrane and in further intracellular compartments (6, 7). These PRRs trigger signaling cascades that ultimately lead to the regulation of transcription to increase the production of intracellular anti-viral restriction factors, cytokines such as interferons (IFNs), or regulatory factors (6, 8–10). Viruses in turn have evolved IFN antagonists to counteract these anti-viral responses (11, 12). In addition to the innate immunity-specific PRR-dependent transcriptional programs, changes in host gene expression of virus-infected cells can be attributed to the massive virus-induced remodeling processes of the infected cell. These include not only cytoskeletal changes, but also alterations in the 3D structure of the genome and the occurrence of cell and DNA damage (13, 14). This implies that many differentially regulated transcripts in virus-infected cells may not have discernible functions and may be the result of cytopathic effects or perturbation of RNA polymerase II function that is affected by various viruses (15).

Despite a wealth of data describing changes in cellular RNA abundance of virus-infected cells (10, 16–19), it remains unclear whether there are conserved patterns in transcriptional responses of virus-infected host cells. In addition, the comparability of existing data sets is limited due to the analysis of different host species, cell types, and experimental variations in sample processing. Moreover, there is limited information on the extent of variations in cellular RNA abundances in cells infected with highly virulent and less virulent viruses.

To address these questions, we used RNA sequencing (RNA-seq) to compare transcriptomic changes in human HuH7 hepatoma cells infected in parallel with several different NSVs. Our large-scale and time-resolved analysis revealed a correlation between the amount of viral RNA in host cells and the number of regulated cellular RNAs. Moreover, we identified a core set of 178 differentially expressed genes (DEGs) that are jointly regulated by all viruses, many of which encode proteins that form a regulatory network.

## RESULTS

### Comparative RNA analysis of NSV-infected cells—overview

**Fig 1:**
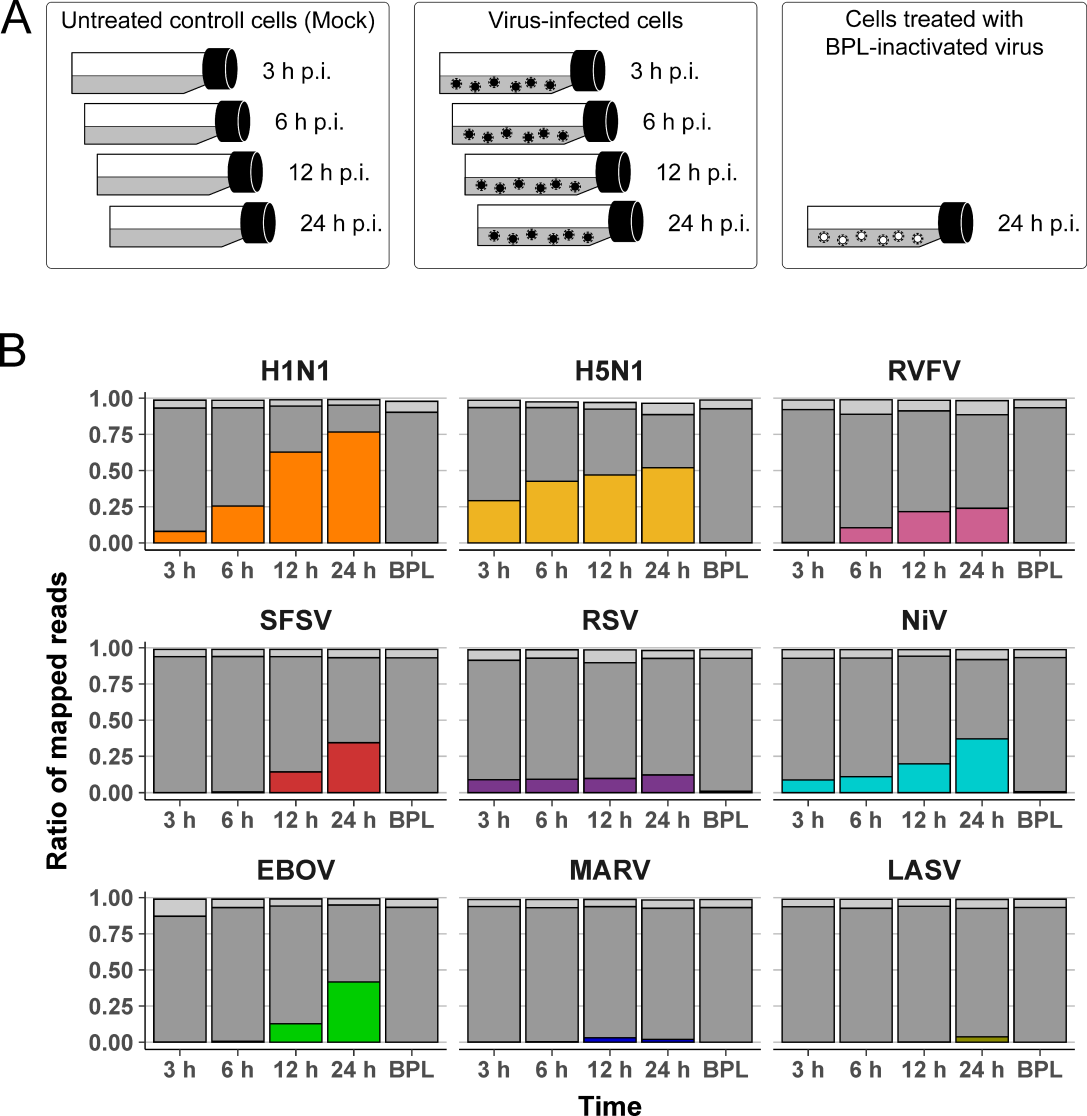
Comparative analysis of RNA expression in NSV-infected cells. (**A**) Visualization of the workflow, infectious virus particles are shown in black, BPL-inactivated virus particles are shown in white. (**B**) Percentage of sequenced reads per sample assigned to either host cell or virus genome. For host sequences, uniquely mapped (dark gray) and multi-mapped (light gray) reads are shown; additional read assignments are given in Table S1. Colored bars show reads aligned to the respective virus genomes. All values are means of the respective replicates. BPL, samples of cells incubated for 24 h with BPL-inactivated virus.

To capture the transcriptional response elicited by a broad spectrum of NSVs, we analyzed members of families with different levels of virulence (Table 1), as defined by the case fatality ratio and severity of symptoms in humans. In particular, these were the orthomyxovirus strains influenza virus A (H1N1; intermediate virulence and H5N1; high virulence) (20, 21), the phenuiviruses Rift Valley fever virus (RVFV; intermediate virulence) (22) and sandfly fever Sicilian virus (SFSV; low virulence) (12), the paramyxoviruses respiratory syncytial virus (RSV; intermediate virulence) (23) and Nipah virus (NiV, high virulence) (24), the filoviruses Ebola virus (EBOV; highly virulent strain Zaire) (25) and Marburg virus (MARV, highly virulent) (26), as well as the Lassa mammarenavirus (LASV, high virulence) (27). As depicted schematically in Fig. 1A, we infected HuH7 cells, a widely used hepatocyte-derived carcinoma line that is permissive to all viruses used in this study (28). Maximal data comparability was ensured by using cells with identical passage number (prepared and distributed by one of the participating laboratories) and the same reagents, as specified in Materials and Methods. A defined number of cells was infected with the different viruses at a predefined multiplicity of infection (MOI) to ensure infection of >80% of the cells. Cells were also incubated with viruses that were previously inactivated with beta-propiolactone (BPL) to distinguish between gene expression changes caused by (i) actively replicating viruses or (ii) inactivated viruses, the latter potentially resulting from plasma membrane interactions, endocytosis, or other mechanisms not directly linked to virus replication. Total RNA was analyzed from two biological replicates by RNA-seq at various time points to monitor gene expression changes at early, middle, and late time points post infection.

**TABLE 1:** List of NSVs used and corresponding genome identifiers

Virus	Strain	PMID	Virulence	Genome
H1N1	A/Giessen/06/2009	29955068	Intermediate (29)	KC620383 –KC620390
H5N1	A/Thailand/1(KAN-1)/2004	29955068	High (30)	CY111595–CY111602
RVFV	ZH548	28457351	Intermediate (31)	NC_014395–NC_014397
SFSV	Sabin	30067170	Low (32)	F. Weber, unpublished data
RSV	A2	32626981	Intermediate (33)	KT992094.1
NiV	Malaysia	30251294	High (34)	NC_002728
EBOV	Orthoebolavirus virus zairense, isolate Mayinga	32080199	High (35)	NC_002549.1
MARV	Orthomarburgvirus marburgense, isolate Musoke	32758690	High (36)	NC_001608.3
LASV	Josiah	36097163	High (37)	NC_004296.1; NC_004297.1

^*a*^ H1N1, H5N1, influenza A virus; RVFV, Rift Valley fever virus; SFSV, sandfly fever Sicilian virus; RSV, respiratory syncytial virus; NiV, Nipah virus; EBOV, Ebola virus; MARV, Marburg virus; LASV, Lassa mammarenavirus.

### Correlation between viral RNA levels and the number of differentially regulated host cell transcripts

Bioinformatic analysis of the sequencing data revealed a comparable number of reads for each sample, and principal component analysis (PCA) confirmed the reproducibility of replicate experiments for different viruses and time points (Fig. S1). As the analyzed RNAs were of both viral and host cell origin, we determined their relative proportion in each sample (Fig. 1B; Table S1). The relative amount of viral RNA compared to host RNA was found to vary widely, ranging between >70% for H1N1 at 24 h and 0.004% for LASV at 3 h. The relative proportions of viral RNA compared to total RNA were very low for LASV and MARV across all time points analyzed. In contrast, the other NSVs showed a continuous increase of viral RNA in the course of infection, as expected for the different stages of active virus replication. The only exception was RSV, which showed an almost maximal amount of viral RNA as early as 3 h post infection. The consistent absence of detectable viral RNA in cells exposed to BPL-treated particles confirmed the effective chemical virus inactivation (Fig. 1B).

**Fig 2:**
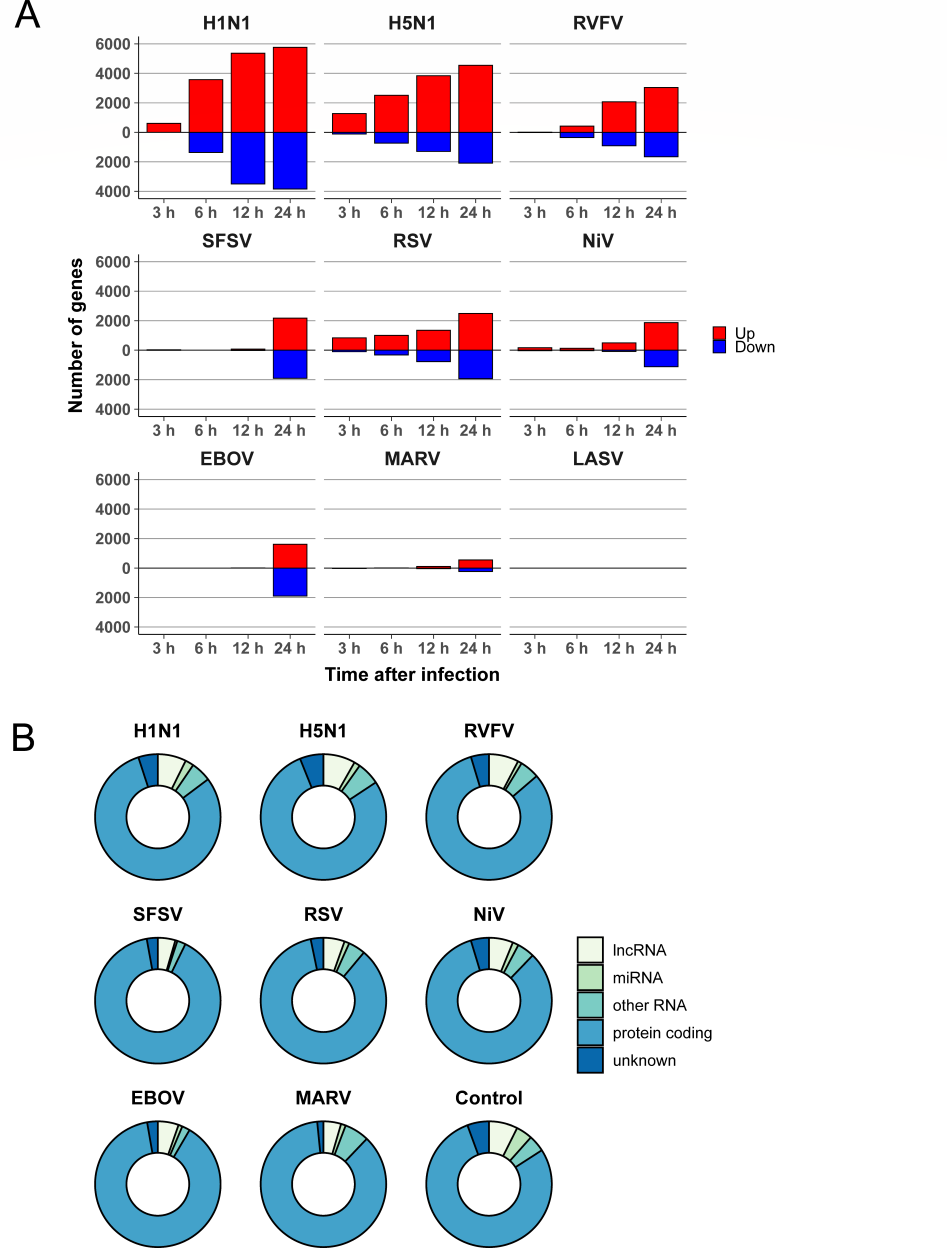
Time-dependent changes in viral and host cell RNAs. (A) RNA expression of the virus-infected host cells was compared with the corresponding uninfected mock control for each time point. Red, up-regulated; blue, down-regulated. (B) Transcripts that showed differential expression for at least one time point were classified into protein-coding mRNAs, lncRNAs, miRNAs, and other RNAs (e.g., pseudogenes and small nuclear RNAs.

To characterize the transcriptomic shift in host cells after infection, we measured DEGs between samples of infected cells and corresponding mock-treated samples using DESeq2 (Fig. 2A). Here, some viruses (H1N1, H5N1, RVFV, and RSV) triggered a time-dependent steady increase in the number of cellular DEGs over the entire time course of the experiment. Other viruses (SFSV, NiV, and EBOV) caused an increase in the number of DEGs only later in the course of infection at 24 h, which might also be attributable to differences in the replication kinetics. In contrast, MARV- and especially LASV-infected cells showed only few DEGs for all time points (Fig. 2A), which also correlated with the low number of viral RNA reads in the sequencing samples (Fig. 1B). Low numbers (<100) of DEGs were previously described in MARV-infected HuH7 cells at 23 h post infection (17). Similarly, exposure of human peripheral blood mononuclear cells or human umbilical vein endothelial cells to LASV resulted in a low number of DEGs (38, 39). The reasons for these limited RNA changes may reflect specifics of the infection strategy and require further studies. For cells infected with SFSV and EBOV, the relative ratio of up- and down-regulated RNAs was comparable. However, in the case of other NSVs, the proportion of up-regulated genes was significantly higher than the proportion of down-regulated genes. Considering all genes that were found to be differentially expressed at one (or more) time point(s) for each virus and classifying the corresponding RNAs, we found that the vast majority of DEGs comprised protein-coding mRNA (78.2%–89.9%), whereas other RNA species such as long non-coding RNAs (lncRNAs), microRNAs (miRNAs), and other RNA species accounted for only a small proportion (7.2%–15.7%) (Fig. 2B).

So far, we used the uninfected mock samples as controls for post infection analysis of DEGs (virus vs mock). In a second step, we calculated DEGs of virus-infected cells compared to cells treated with BPL-inactivated virus as a control (virus vs BPL-control) to estimate the extent of possible effects of inactive virus on RNA abundance. Thus, the intersection of these two comparisons revealed all genes that were differentially expressed in virus-infected cells compared to both (i) the untreated control and (ii) cells treated with BPL-inactivated virus (Fig. S2, shaded). This intersection is comparatively large for each of the viruses analyzed, suggesting that the impact of inactivated viruses on cells is comparatively small. Therefore, the comparisons of virus-infected cells with the corresponding untreated mock samples were used for all subsequent analyses of DEGs in this study.

### Advanced data analysis and visualization with the open access web application ADVICER (the Analysis Dashboard for Virus-Induced Cell Response based on RNA-seq data)

We have targeted the transcriptional response of human host cells to nine different viruses at four distinct time points each. To make the DESeq2 results of this extensive study accessible to everyone, we have implemented the web-based software application ADVICER (https://advicer.computational.bio). This software allows analysis and visualization of the individual data sets, which can be downloaded in tabular or graphical form. The web interface features tabs enabling access to (i) DEGs at individual time points after infection for each virus (Volcano plot or Bland–Altman [MA] plot), (ii) comparisons of gene expression data from the different time points per virus (Venn diagrams, UpSet plots) with the option to download the results as Excel files or heat maps, (iii) a search for specific genes and comparisons of their expression over time for the different virus infections (graphs with kinetics as well as bar charts), and (iv) comparisons of gene expressions triggered by the different viruses at any chosen time point (UpSet plots, Excel files, heat maps). The different functionalities of the application are visually summarized in Fig. S3. These different features of the ADVICER application enable users to generate tables including links to NCBI, compare individual time points and time courses, explore commonly regulated genes, and create heat maps. Various options for customization allow users to address highly specific inquiries tailored to their needs.

### Identification of the common host cell response

**Fig 3:**
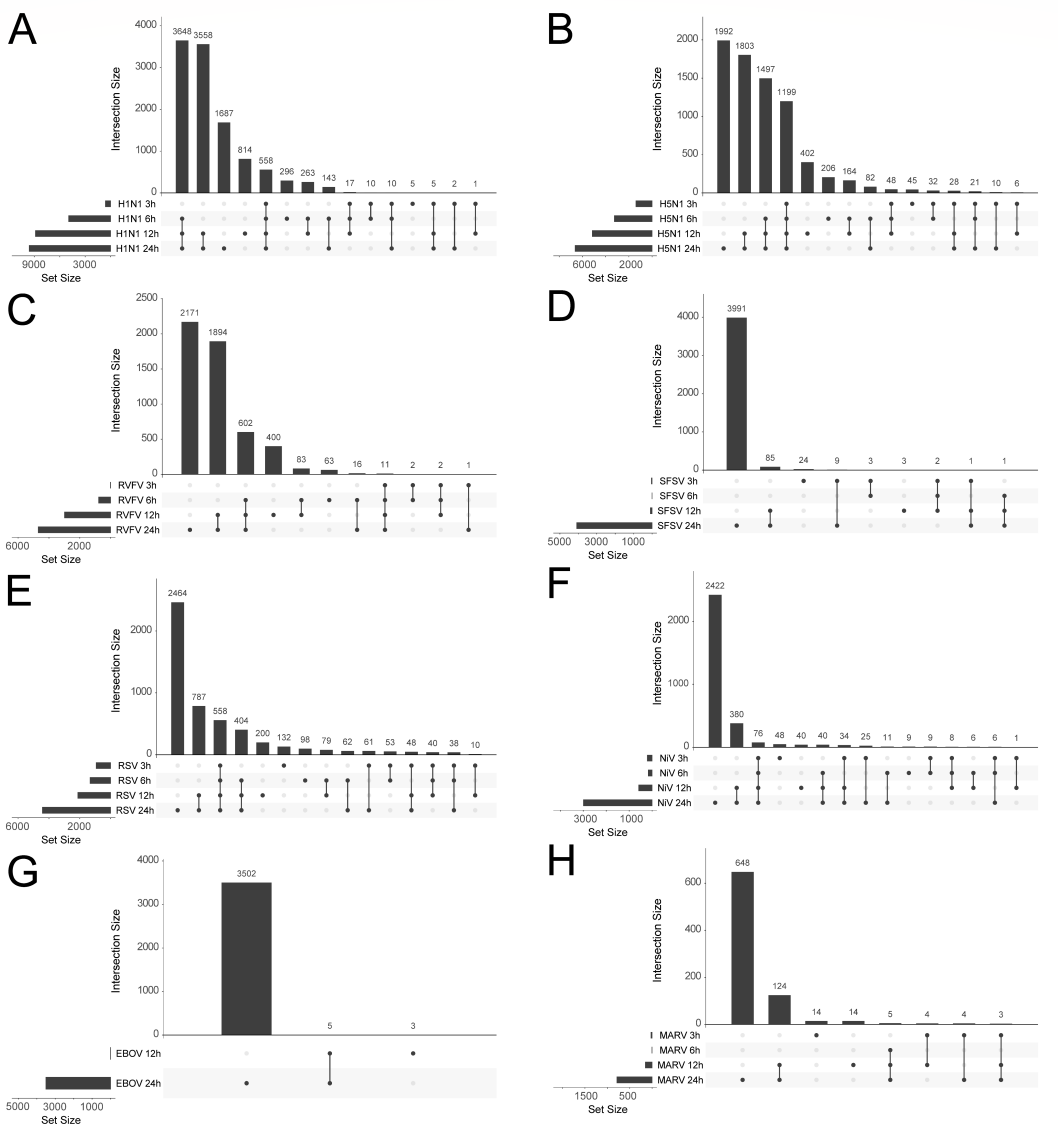
Time-resolved analysis of DEGs for individual infections. The UpSet plot shows all intersections of genes that were differentially expressed at different time points. The top bar graph shows the intersection sizes. The left bar graph shows the size of the gene sets, both graphs are ordered by the set size. Black dots and lines indicate gene sets that are part of a subgroup.

**Fig 4:**
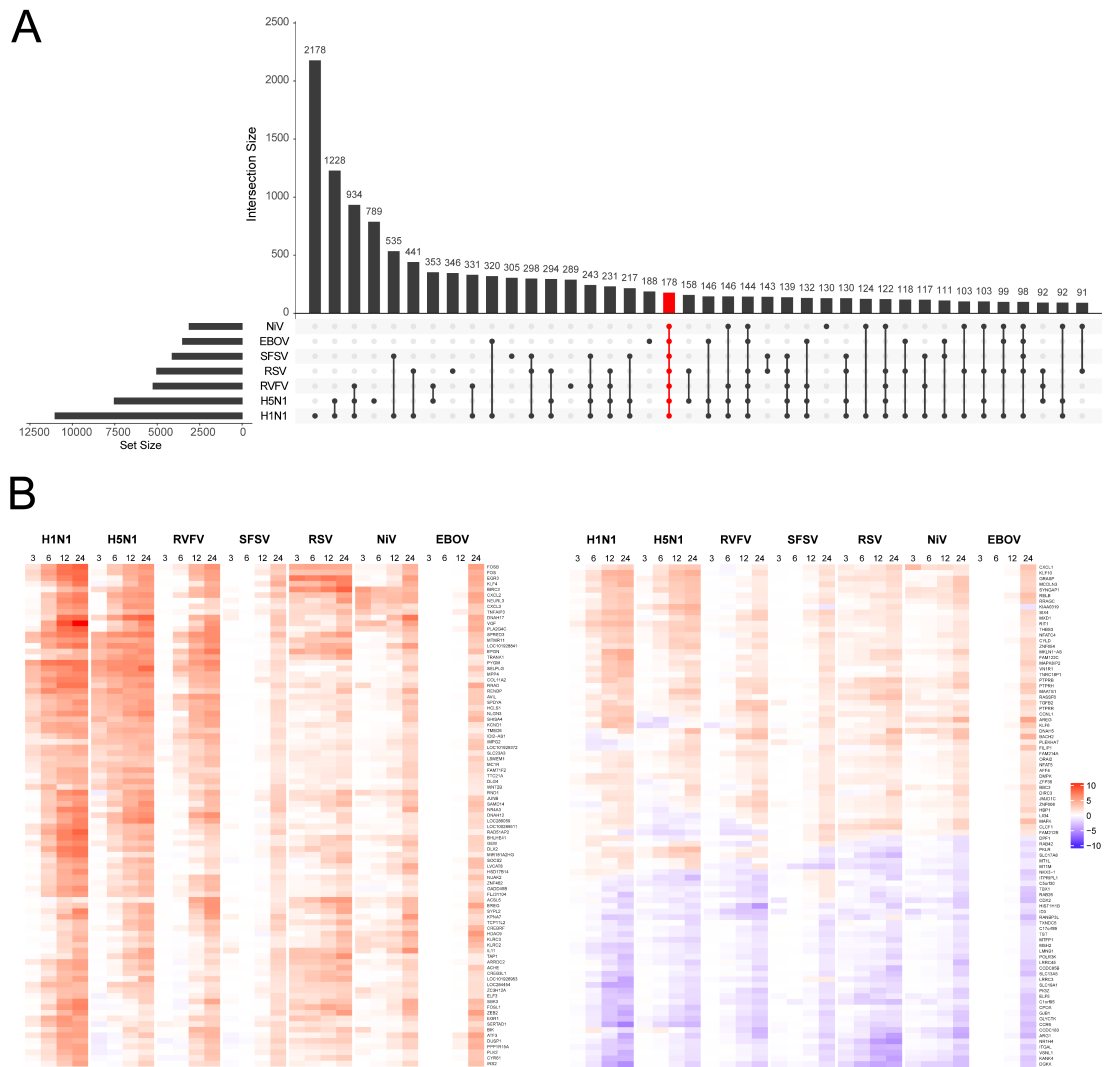
Identification of the common gene response to NSV infections. (**A**) The UpSet plot shows all intersections of genes that were differentially expressed (up or down) in at least one time point for one or more viruses. The top bar graph shows the intersection sizes. The left bar graph shows the size of the gene sets, both graphs are ordered by the set size. Black dots and lines indicate gene sets that are part of a subgroup. The common set is shown in red. (**B**) The heat map shows the expression level of genes commonly differentially expressed in at least one time point. Four different time points per gene are indicated, colors correspond to log2 fold changes.

As the different viruses trigger changes in gene expression within the host cells with specific kinetics (Fig. 2A), we visualized DEG changes by UpSet plots to depict the intersections and set relationships among the different time points for each virus (Fig. 3). For the virus group (SFSV, NiV, EBOV, and MARV) in which DEGs were mainly restricted to later time points post infection, very little overlap of DEGs was observed at early time points post infection. In contrast, in cells infected by one of the viruses that triggered a steady increase of DEGs, only a small proportion of DEGs was exclusively identified for one particular time point (Fig. 3). For identification of the core transcriptional response, MARV and LASV samples were excluded due to their low number of regulated transcripts. We considered all DEGs that were regulated by the different viruses during at least one time point. This analysis revealed a common set of 178 host RNAs (Fig. 4A). Of this core set, 165 genes (92%) are known to be protein-coding mRNAs. A heat map of the regulated transcripts showed that the majority of the core DEGs was up-regulated during virus infection (Fig. 4B).

### Identification of transcription factor networks mediating the common host response

**Fig 5:**
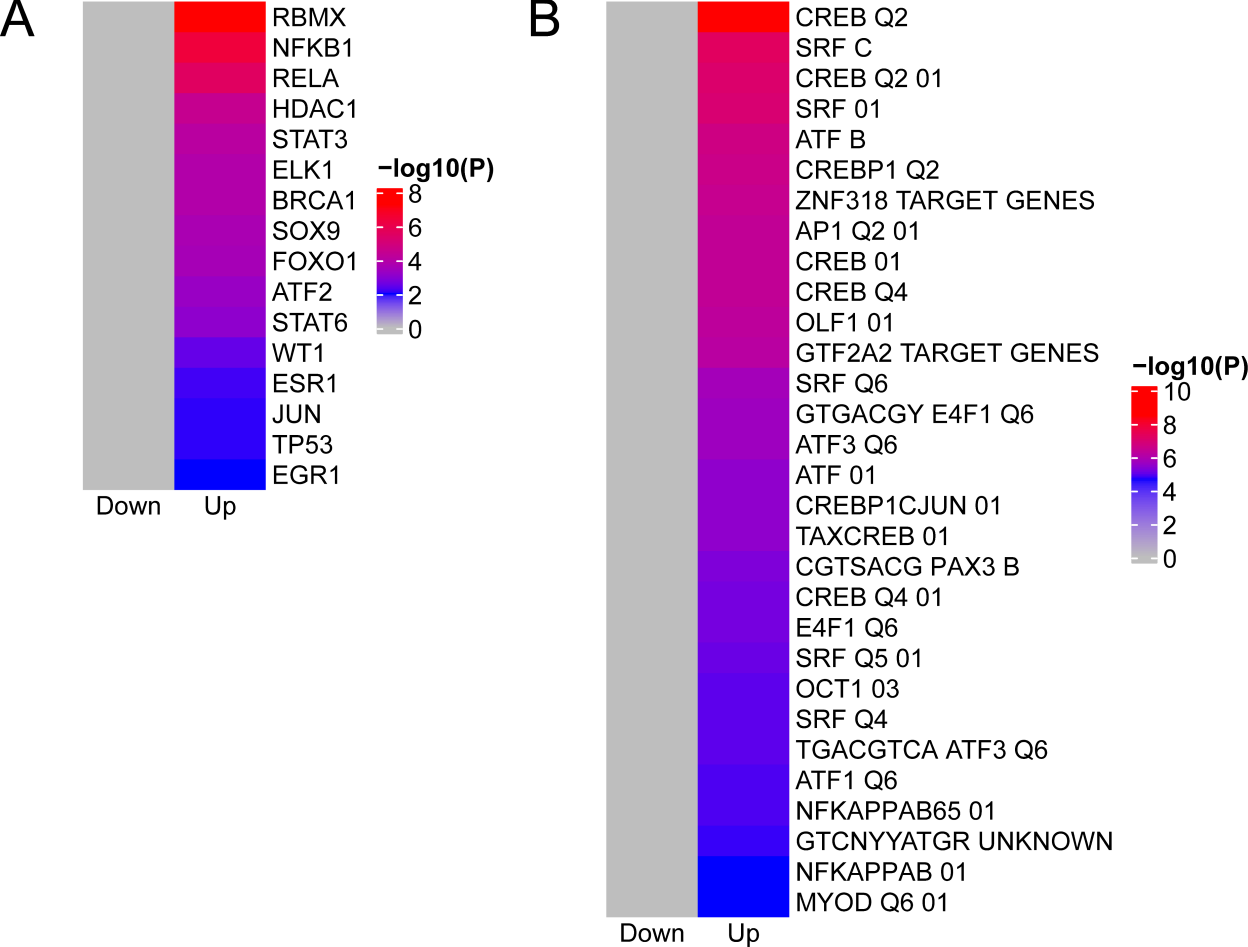
*In silico* prediction of potential transcription factors mediating different RNA expression of the core set of DEGs. (**A**) Potential transcription factors or transcriptional coregulators were identified by the TRRUST. This is a database, in which transcription factor (TF)-target interactions were identified based on sentence-based text mining of PubMed articles. (**B**) Candidate transcription regulators were identified using the molecular signature database MSigDB. Suffixes of transcription factor names represent IDs that allow discrimination of multiple motif entries of a factor. Heat maps depict the negative decadic logarithm of the adjusted *P*-value.

To identify candidate transcription factors contributing to the regulation of the core set of DEGs, we used an over-representation analysis strategy conducted with Metascape to compare the genes to two different databases. First, we used the TRRUST database, which contains information about interactions between transcription factors and corresponding target genes (40). In this approach, no significant association between down-regulated core DEGs was detected, suggesting that reduced RNA levels may be independent of specific transcriptional regulatory factors, whereas up-regulated transcripts were associated with several regulators of gene expression (Fig. 5A). In an orthogonal approach, we identified candidate transcription factors using the MSigDB Transcription Factor Targets (TFT) collection, a curated database that provides information about transcription factor binding sites at target genes (https://www.gsea-msigdb.org/gsea/msigdb/collections.jsp). This approach also identified a number of different transcription factors that only target the up-regulated DEGs (Fig. 5B). Both approaches independently identified members of the NF-κB and AP-1 family of transcription factors, and in support to their functional relevance, many of the core DEGs are known target genes of these transcription factors (e.g., CXCL2, CXCL3, and IL11) (41). While NF-κB and AP-1 family members interact with each other and frequently cooperate in the regulation of gene expression (42), they can form bigger transcription factor networks to orchestrate inflammatory gene expression upon association with STAT (signal transducer and activator of transcription), IRF (interferon regulatory factor), CREB (cAMP response element-binding protein), and ATF (activating transcription factor) (43, 44), of which several representatives have been identified in the over-representation study, except for IRF (Fig. 5A and B). Interestingly, several of the identified or closely related transcription factors are themselves up-regulated and part of the core set of DEGs (ATF3, CREB3L1 and CREBRF, EGR1 and EGR3, FOS, FOSB, FOSL1 and JUNB, HDAC9, RELB), further supporting their role as direct regulators of the common transcriptional response of cells infected with NSVs.

### Characterization of the common transcriptional host cell response

**Fig 6:**
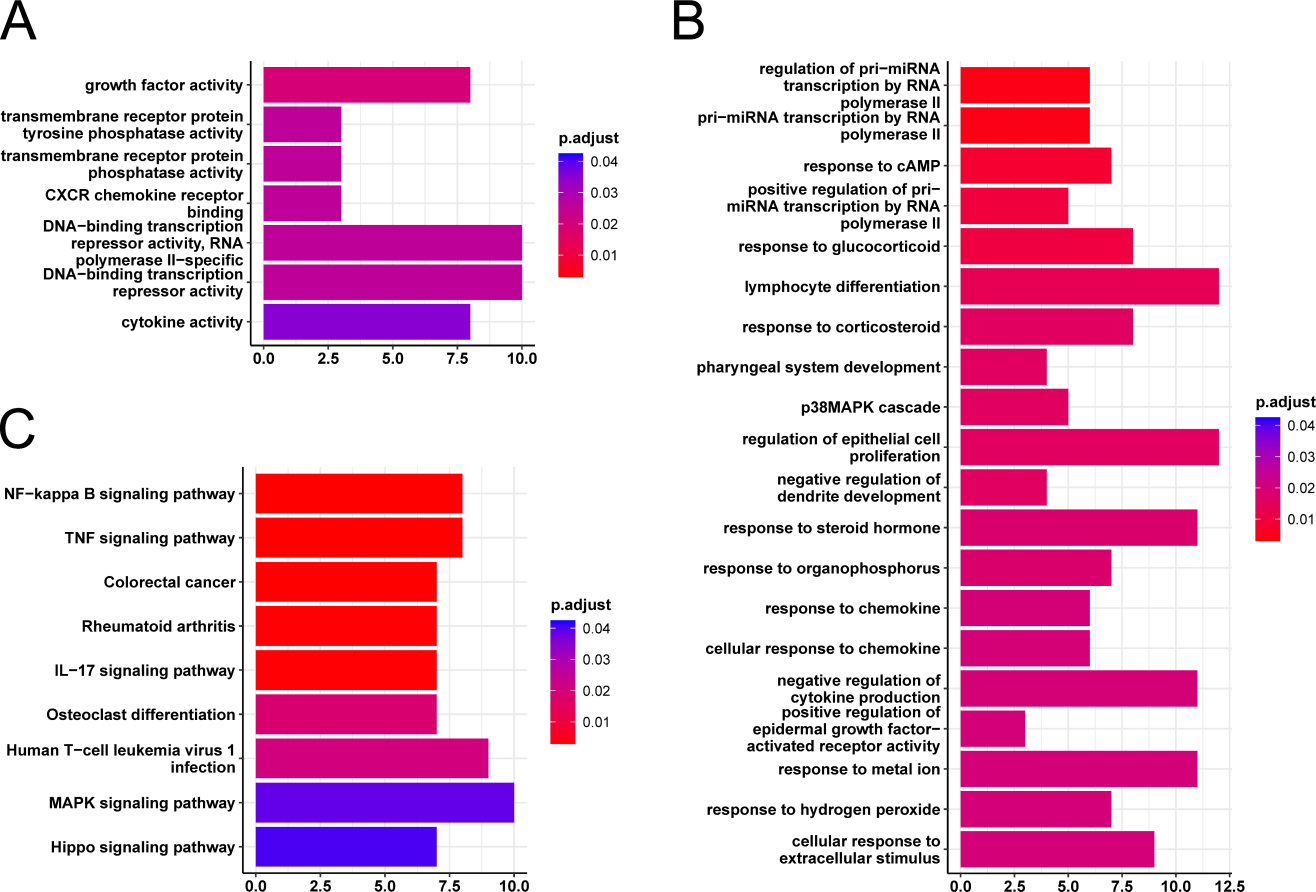
Over-representation analysis of the core DEGs. Genes were analyzed by plotting on key GO terms for molecular function (**A**) and biological process (**B**) and KEGG pathways (**C**). Significance of enrichment is indicated by adjusted *P*-values and bar colors.

**Fig 7:**
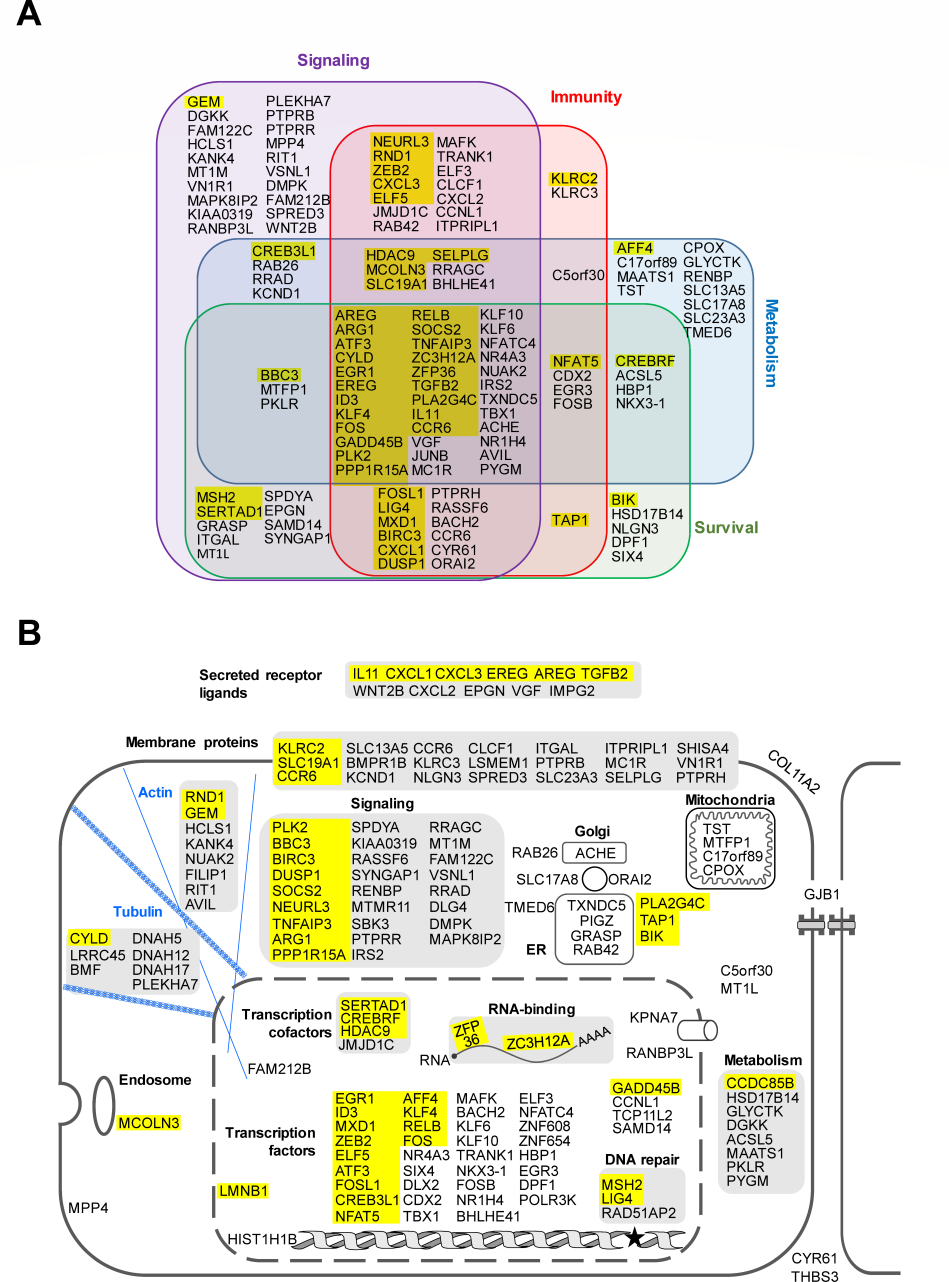
Functional and spatial organization of proteins encoded by the core DEGs. (**A**) Proteins with known function in regulating the outcome of infections with RNA or DNA viruses (yellow color) or the indicated biological processes. (**B**) The intracellular localization of the core DEG-encoded proteins was determined using the protein atlas (https://www.proteinatlas.org/) and is shown in the schematic cell. Components of the cytoskeleton are highlighted in blue.

The set of 178 host RNAs was subjected to an over-representation analysis of functional annotations using GO (gene ontology) and KEGG (Kyoto Encyclopedia of Genes and Genomes) databases. The analysis for GO class “molecular function” showed enrichment of annotations related to DNA-binding factors and transcription, but also to cytokine activity and signaling (Fig. 6A); the analysis for “biological process” showed the involvement of proliferation/differentiation, but also of signaling events and cell stress (Fig. 6B). The KEGG analysis revealed a major involvement of inflammatory events and components of the innate immune system (Fig. 6C). We also performed a functional categorization of the protein-coding genes of the core set, confirming the importance of innate immunity and signaling, and also showing the relevance of cell death processes and metabolic regulation (Fig. 7A). Most of the proteins are known to participate in more than one of these processes.

**Fig 8:**
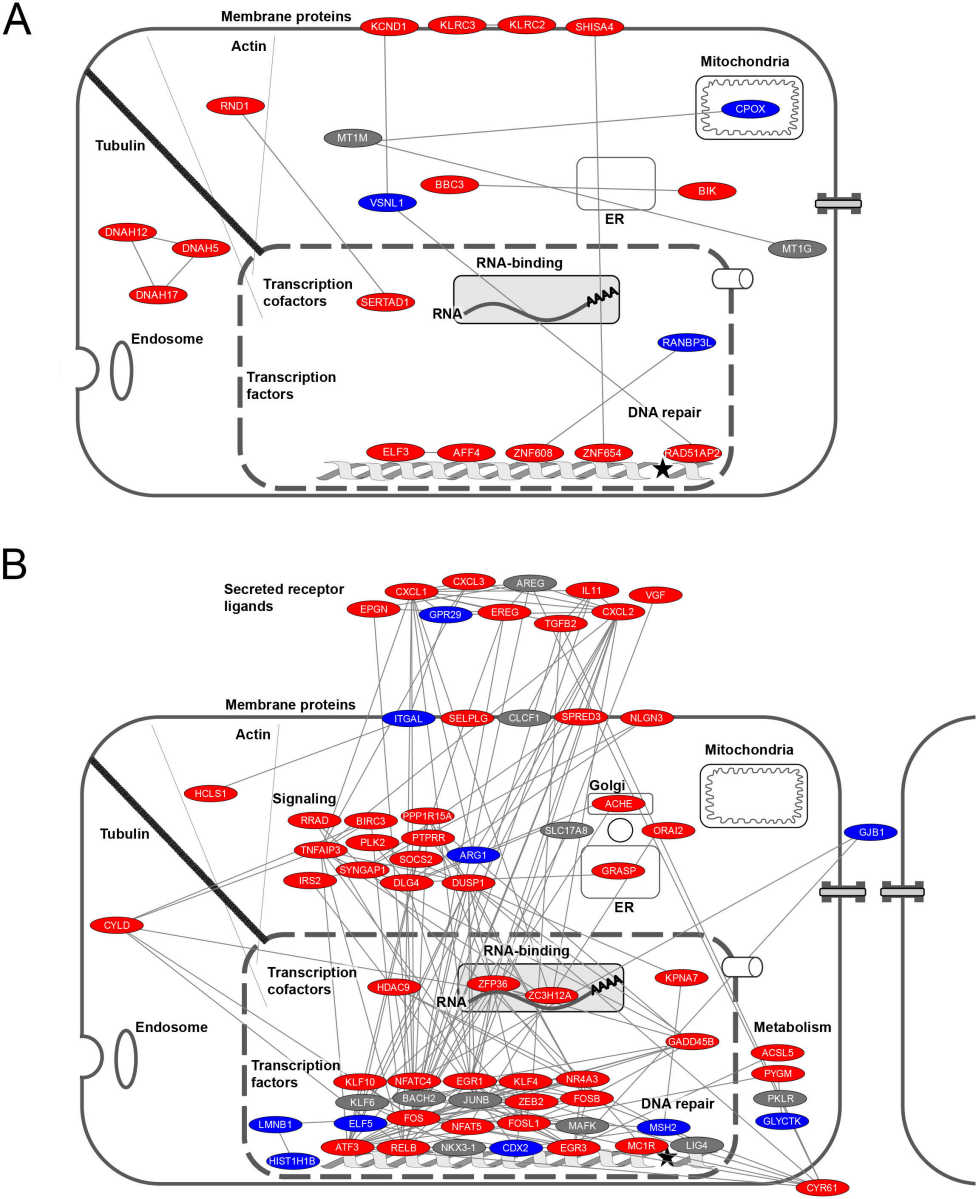
Network analysis of proteins encoded by the core DEGs. The proteins were analyzed for known and predicted interactions using the STRING database (version 11.5) as indicated. While (**A**) shows several smaller networks involving two or three interactions, (**B**) displays the main network with >3 protein/protein interactions. The regulation of transcripts is indicated by different colors (up, red; down, blue; no uniform regulation, gray).

We compared the protein-coding RNAs of the core set with published data to estimate the importance of these genes in viral infections and found that 48 of the 165 protein-coding RNAs (29%) have a previously documented pro- or anti-viral role in RNA or DNA virus infections (see Fig. 7A), whereas such functions have not yet been described for the remaining proteins. The graphical representation of cellular protein localization shows that few proteins are exported to the extracellular space (Fig. 7B). The largest groups are formed by nuclear transcription factors and cytosolic signaling proteins, in agreement with the GO analysis displayed in Fig. 6. While the representation of mediators of inflammation is not unexpected, this analysis also suggested that a number of proteins encoded by the core DEGs are functionally or physically associated with components of the cytoskeleton, supporting previously published data on the central role(s) of the actin and microtubule cytoskeleton in the viral life cycle (45–47). In addition, further protein groups have functions in the classical secretory pathway or metabolic regulation that are both known to be required for virus replication (48, 49). The STRING database was used to reveal known and predicted interactions between the coregulated proteins. This analysis revealed several smaller networks (Fig. 8A) and one large regulatory network involving many of the proteins encoded by the coregulated mRNAs (Fig. 8B), suggesting their functional cooperation in the host cell response to infection with NSVs.

## DISCUSSION

Here, we identified time-dependent changes in the transcript abundance in human HuH7 cells infected in parallel with nine different NSVs. Our data are publicly accessible via the tailor-made web-based application ADVICER and can be visualized and downloaded in a variety of different ways. While the open access ADVICER web app allows all sorts of tailored comparisons, this study focused on the analysis of the core set of 178 (mostly up-regulated) DEGs. The proteins encoded by these transcripts are involved in the regulation of immune response, signaling, cell survival, metabolic processes, and the cytoskeleton. We suspect that a number of transcripts are also regulated by indirect events due to virus infection-related cell damage. It remains to be investigated if the observed changes in RNA levels are due to alterations in RNA stability and/or their *de novo* synthesis. Candidate transcription factors participating in mRNA synthesis were identified by *in silico* prediction. Two different approaches suggested a relevant role of NF-κB and AP-1 transcription factors for expression of many of the up-regulated genes, consistent with the prominent role of these transcription factors in the innate immune response and viral infections (6, 50). Interestingly, in response to viral infection, a significant number of NF-κB/AP-1 family members and their collaborating factors from the ATF and CREB families show transcriptional upregulation, thereby potentially contributing to an additional level of their activation. The *in silico* analysis did not identify any transcription factor controlling the expression of the down-regulated transcripts, so that the molecular mechanisms mediating this effect remain unclear. One possible mechanism could involve RNA-binding proteins like RBMX, which was identified through a computational approach (Fig. 5A), or target genes encoding RNA-binding proteins such as ZFP36 and ZC3H12A (Fig. 7B). Virus infections can not only lead to profound changes in the host cell RNA landscape by causing changes in RNA levels, but also affect polyA length, transcription start site selection, RNA editing, splicing, and RNA secondary structures (51–54). Moreover, segmented NSVs like H1N1, H5N1, RVFV, SFSV, and LASV cleave host RNAs in order to obtain primers for their own mRNA transcription (55), which could lead to deprivation of specific transcript populations.

Although some of the DEGs have no known function, transcript changes may be of great predictive value for the severity of infection and disease outcome, as, for example, described for infections with dengue or Ebola virus (56, 57). To identify potentially relevant transcripts, we used a highly standardized infection model, followed by massive parallel sequencing to avoid biases that are regularly picked up upon comparison of data sets from public databases. This strategy allowed the identification of a common core set of coregulated genes in NSV-infected cells. As transcriptome changes depend on the cell type and show a cell-to-cell variability (58, 59), it will be informative to perform further large-scale RNA-seq experiments using an extended set of viruses in additional cell types. These analyses can show whether the core set identified in the current study is also regulated in other systems. As viral infections also lead to changes in decay and synthesis of proteins by regulation of host translation factors and ribosome availability (60), which can ultimately lead to “host cell shut-off” (61), we assume that not all changes observed at the RNA level are necessarily reflected at the protein level which will require proteome-wide analysis. While the virulence of NSVs used in this study had no discernible impact on the number of DEGs (see, e.g., the substantial difference between the equally virulent EBOV and MARV), we observed a clear correlation between the amount of viral RNA and the number of regulated host cell transcripts.

In the set of common genes, we specifically investigated the role of protein-coding mRNAs, as miRNAs and lncRNAs employ various mechanisms to regulate a large number of different targets (62, 63). Interestingly, the set of common genes is highly enriched in known regulators of infection with RNA or DNA viruses (47 of 178 total RNAs); for details, see Table S2. It will therefore be interesting to study the role of the identified transcripts for a potential role in virus replication and viral restriction in the future. Most of the proteins (89 from 165) encoded by the core DEGs form one large and several smaller networks, raising the possibility that components of this network play a regulatory role in viral infection. The organization of signaling pathways into large networks allows integration and coordination of different inputs and dynamic shaping of adequate output responses. This allows the reduction of signaling noise, while increasing robustness, dynamics, and signal amplification (64, 65). Such dense network hubs are considered as ideal perturbation entry points for small molecule effectors that can be used experimentally or therapeutically (66). It will thus be relevant for future experiments to perturb the genes of these signaling hubs either alone or in combination and to study their potential function in virus replication. This might enable the precise targeting of key host factors using pharmacological or PROTAC-based approaches to enable new strategies for anti-viral intervention that could potentially even interfere with infection by different viruses.

## MATERIALS AND METHODS

### Cells and viruses

HuH7 human hepatoma cells (JCRB Cell Bank) were cultured in Dulbecco’s Modified Eagle Medium supplemented with 10% (vol/vol) fetal bovine serum (FBS) and grown at 37°C in the presence of 5% (vol/vol) CO_2_. As the infection experiments were run by several laboratories, maximal comparability of data sets was ensured by using identical cells at the same passage number and identical reagents including cell culture media, plasticware, FBS, and RNA isolation kits. The virus stocks were produced in appropriate cells, followed by concentration via ultracentrifugation or Amicon centrifugation (Fig. S4A). Cells were infected with nine different viruses listed in Table 1 at an MOI of 3 to achieve infection of more than 80% of the cell population, as confirmed by parallel immunofluorescence analysis (Fig. S4). The infection experiments were performed in parallel in three biosafety laboratories (BSL-4, BSL-3, and BSL-2) with the appropriate containment required for the respective virus(es) under study.

### Sample collection and RNA sequencing

Samples were taken at 3 h, 6 h, 12 h, and 24 h after infection. In addition, two different types of control samples were prepared: (i) samples from uninfected (mock-treated) cells and (ii) samples from cells exposed to BPL-inactivated viruses (only 24 h post infection), as schematically shown in Fig. 1A. Total RNA was isolated using the Qiagen RNA Kit. The rRNA depletion and library preparation was completed using Illumina’s TruSeq stranded total RNA kit, and sequencing was performed on an Illumina HiSeq 4000 instrument (single read, 150 bases) (Illumina, San Diego, California) at the EMBL in Heidelberg (Germany). For each time point, two biological replicates were sequenced, except for RVFV at 24 h after infection, where only one replicate could be sequenced due to the cytopathic effect. RNA-seq data sets contained information on host cell and viral RNAs. An automated workflow for the evaluation of human transcriptome and viral RNA genome data was applied, including RNA-seq quality control with FastQC version 0.11.9 (https://www.bioinformatics.babraham.ac.uk/projects/fastqc/) as well as adapter removal and data trimming with fastp version 0.20.1 (67).

### Sequence data processing

Preprocessed reads were mapped to the human genome with STAR version 2.7.5 c (68) using the hg38 UCSC gene annotation. The human genome and gene annotation were downloaded from Illumina’s iGenome homepage (https://emea.support.illumina.com/sequencing/sequencing_software/igenome.html). For RNA samples derived from cells infected with viruses, the corresponding viral genome was added to the human reference prior to read mapping as an additional “chromosome.” Publicly available viral genomes listed in Table 1 were used as reference for all analyses. Gene-specific read counts based on hg38 UCSC gene annotations were extracted using featureCounts from the Subread package version 2.0.1 (69). Resulting read counts were imported into R (Team, 2015) and normalized using DESeq2 version 1.30.1 (70). DEGs were detected upon comparison between the uninfected mock sample and the infected sample using DESeq2. To determine the effects of virus replication on infectivity, samples from cells infected with virus for 24 h were compared with samples exposed to BPL-inactivated virus for 24 h.

### Visualization and enrichment analysis

For visualization and ranking, log2 fold change (LFC) shrinkage was performed using lfcShrink from DESeq2 with the ashr (71) method. PCA was performed with the plotPCA function from DESeq2. Venn diagrams were created with the R package VennDiagram version 1.7.1 (72). UpSet plots were generated with the R package UpSetR version 1.4.0 (73) using intersection ordering by frequency. Functional enrichment analyses of GO terms (74, 75) and KEGG (76–78) pathways were calculated using clusterProfiler version 3.18.1 (79). For these analyses, gene symbols were converted into Entrez gene identifiers with the function bitr from clusterProfiler and the org.Hs.eg.db annotation package version 3.12.0 (https://bioconductor.org/packages/release/data/annotation/html/org.Hs.eg.db.html). To compare differential gene expression of different viruses, a list of genes that were significantly differentially expressed at one or more time point(s) was generated for each virus. A gene was considered significantly differentially expressed if the absolute log2 fold change was >1, the adjusted *P*-value was <0.05, and at least one sample had a normalized read count ≥10. The STRING database (version 11.5) (80) was used to reveal known and predicted interactions between the proteins encoded by the commonly regulated interactors (full string network, medium confidence 0.4). Visualization of the workflow displayed in Fig. 1A was done with Biorender.

### Interactive application

All DESeq2 data sets and their comparative visualizations can be accessed and further studied in detail using ADVICER, the Analysis Dashboard for Virus-Induced Cell Response based on RNA-seq data. ADVICER is an R Shiny application deployed via a Docker container in a Kubernetes cluster at the de.NBI cloud in Giessen, available at https://advicer.computational.bio. The DESeq2 result tables are used as input and can be downloaded via the application. The genes are classified as significantly differentially expressed as described above, and the user can change the LFC threshold for each analysis individually if needed. MA plots and Volcano plots are generated interactively by using the R package plotly (https://plotly-r.com/index.html), and users can download the plots. Venn diagrams and UpSet plots are generated with the R package upsetjs (https://CRAN.R-project.org/package=upsetjs). Subsets from Venn diagrams and UpSet plots can be printed as a data table using the DT package (https://CRAN.R-project.org/package=DT) and downloaded in csv or xlsx format. The data tables can also be plotted as a heat map with InteractiveComplexHeatmap (81). The time-resolved gene expression is plotted based on ggplot2 (https://ggplot2.tidyverse.org/).

### Transcription factor analysis

Transcription factor analysis was conducted using the web-based tool Metascape version 3.5.20230101 (82) (https://metascape.org/) with the TRRUST (40) and MSigDB C3 TFT subcollection (83–85) databases. TRRUST is a database, in which TF-target interactions were identified based on sentence-based text mining of more than 10,000 PubMed articles. TFT is a collection of genes that were compiled based on the presence of corresponding recognition motifs in defined intervals around the transcriptional start sites and they thereby independently provide evidence for a direct regulatory role of the respective transcription factor. The Metascape analysis was performed by uploading the list of core DEGs to the web interface and running the express analysis with default parameters. The result tables of the TRRUST and the MSigDB Transcription Factor Targets enrichment analyses were imported into R and used to create custom heat maps with the package ComplexHeatmap (86). The *P*-value for the transcription factor analysis describes the result of a hypergeometric test which is corrected by the Benjamini-Hochberg procedure. It represents the probability that the number of genes observed to be associated with a certain transcription factor (by comparison to TRRUST and MSigDB transcription factor databases) occurred just by chance (randomly).

## Data Availability

The RNA-seq data generated in this study have been deposited in the Short Read Archive (SRA) of the National Center for Biotechnology Information (NCBI) under the BioProject ID PRJNA1074963. The app can be accessed via: https://advicer.computational.bio/.

## SUPPLEMENTAL MATERIAL

Supplemental figures jvi.00935-24-s0001.pdf Fig. S1 to S4.

Table S1 jvi.00935-24-s0002.xlsx Read counts of cell- and virus-derived RNAs.

Table S2 jvi.00935-24-s0003.docx 47 genes regulated by all viruses with a known function in infection by RNA or DNA viruses.

ASM does not own the copyrights to Supplemental Material that may be linked to, or accessed through, an article. The authors have granted ASM a non-exclusive, world-wide license to publish the Supplemental Material files. Please contact the corresponding author directly for reuse.
