# Supplementary material for: Distinct negative-sense RNA viruses induce a common set of transcripts encoding proteins forming an extensive network
Source: J Virol. 2024 Sep 16;98(10):e00935-24. doi: 10.1128/jvi.00935-24 (PMC11494938; doi:10.1128/jvi.00935-24)
Supplement: Supplemental figures — Fig. S1 to S4. [file jvi.00935-24-s0001.pdf]

Supplementary Figures

Distinct negative-sense RNA viruses induce a common set of transcripts encoding proteins forming an extensive network

Nina Hofmann, Marek Bartkuhn, Stephan Becker, Nadine Biedenkopf, Eva Böttcher-Friebertshäuser, Karina Brinkrolf, Erik Dietzel, Sarah Katharina Fehling, Alexander Goesmann, Miriam Ruth Heindl, Simone Hoffmann, Nadja Karl, Andrea Maisner, Ahmed Mostafa, Laura Kornecki, Helena Müller-Kräuter, Christin Müller-Ruttloff, Andrea Nist, Stephan Pleschka, Lucie Sauerhering, Thorsten Stiewe, Thomas Strecker, Jochen Wilhelm, Jennifer D. Wuerth, John Ziebuhr, Friedemann Weber, M. Lienhard Schmitz

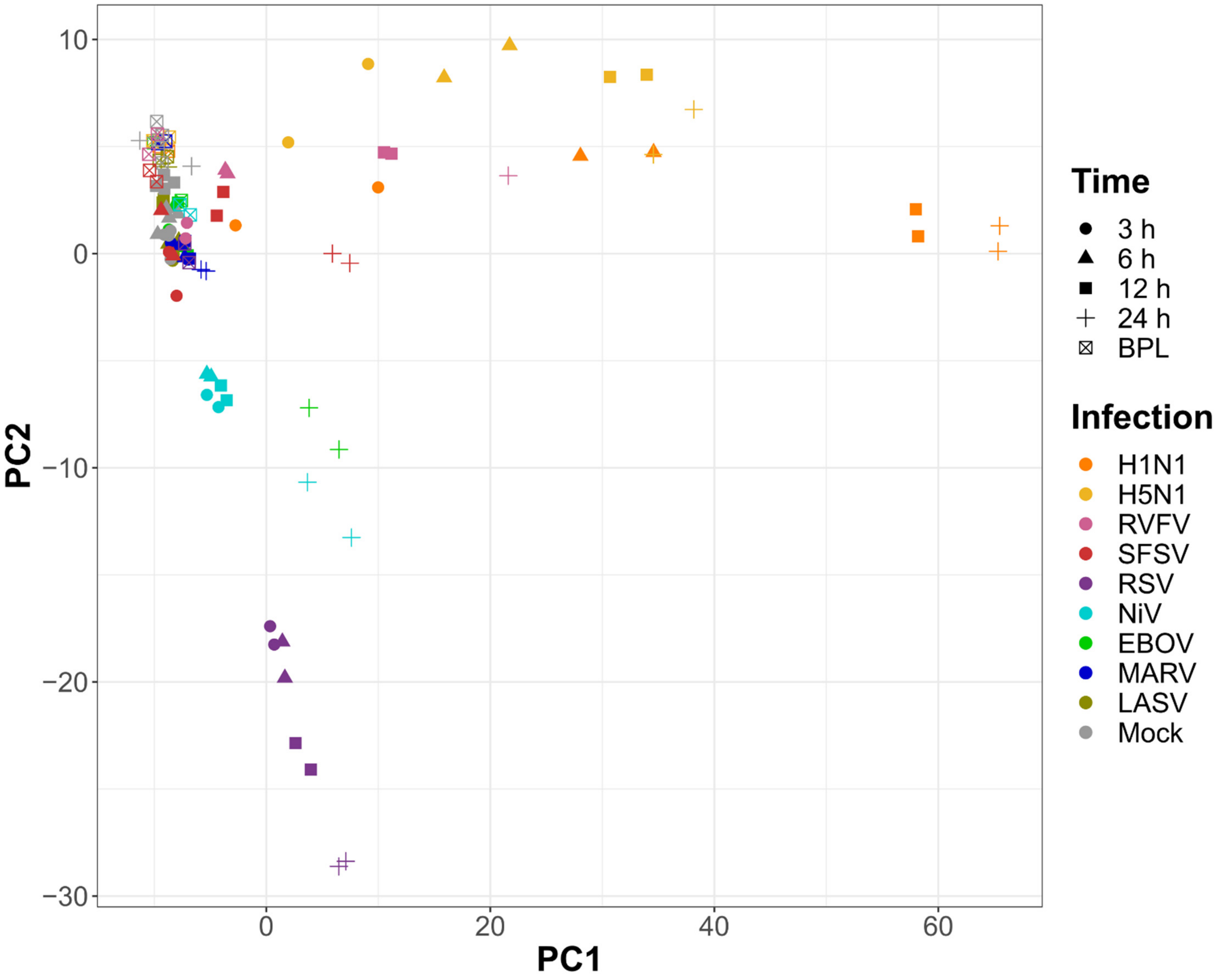

Suppl. Fig. S1. PCA for infections with different viruses and varying sampling time points. The plot shows the first two components of the PCA. The color of the dots represents the treatment of the samples. The shape of the dots indicates the time point the samples were taken. The analysis is based on DESeq2 normalized read counts per gene.

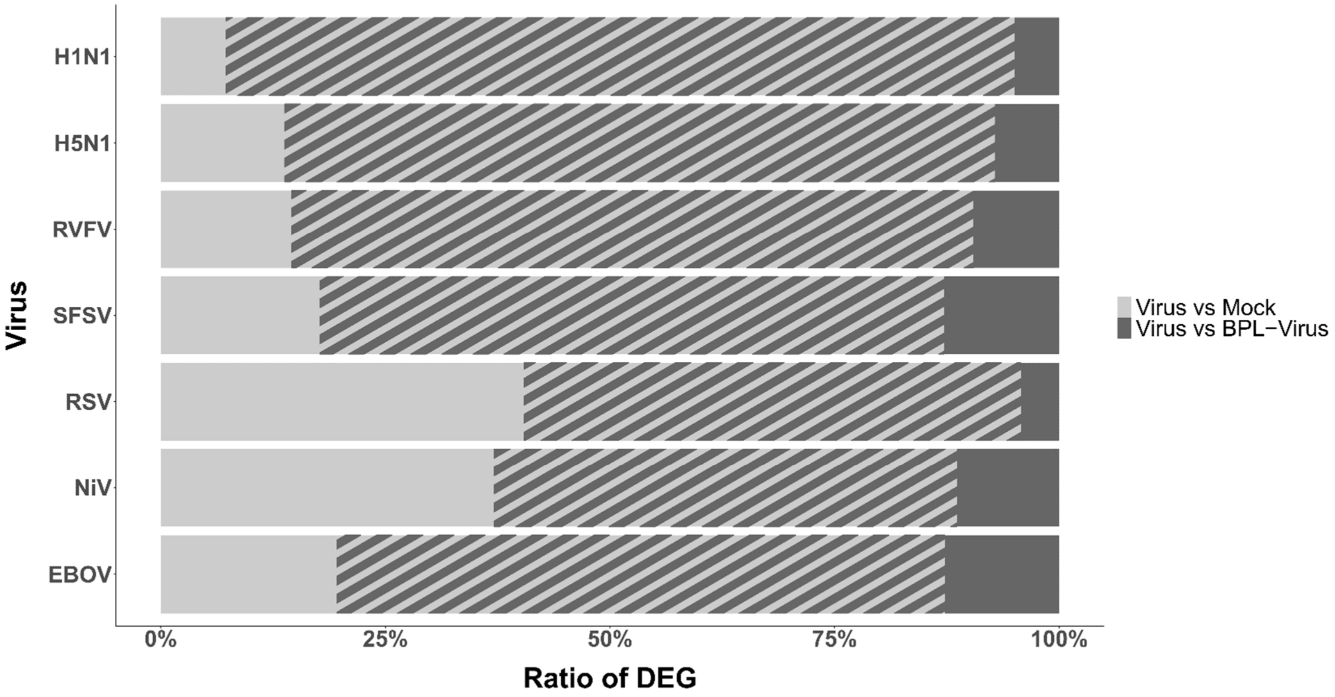

Suppl. Fig. S2. Effects of virus inactivation on RNA expression. Cells remained untreated or were infected with viruses or BPL-treated viruses. After sequencing, the DEGs were calculated for virus-infected cells compared to either untreated cells (light gray) or cells treated with BPL-inactivated virus (dark gray), in each case for the time point 24 h. The overlap of these two comparisons shows genes differentially expressed in virus-infected cells compared to both controls (shaded gray). DEG = ± LFC > 1, adjusted *P* value < 0.05, ≥ 10 mapped genes in at least one sample.

A

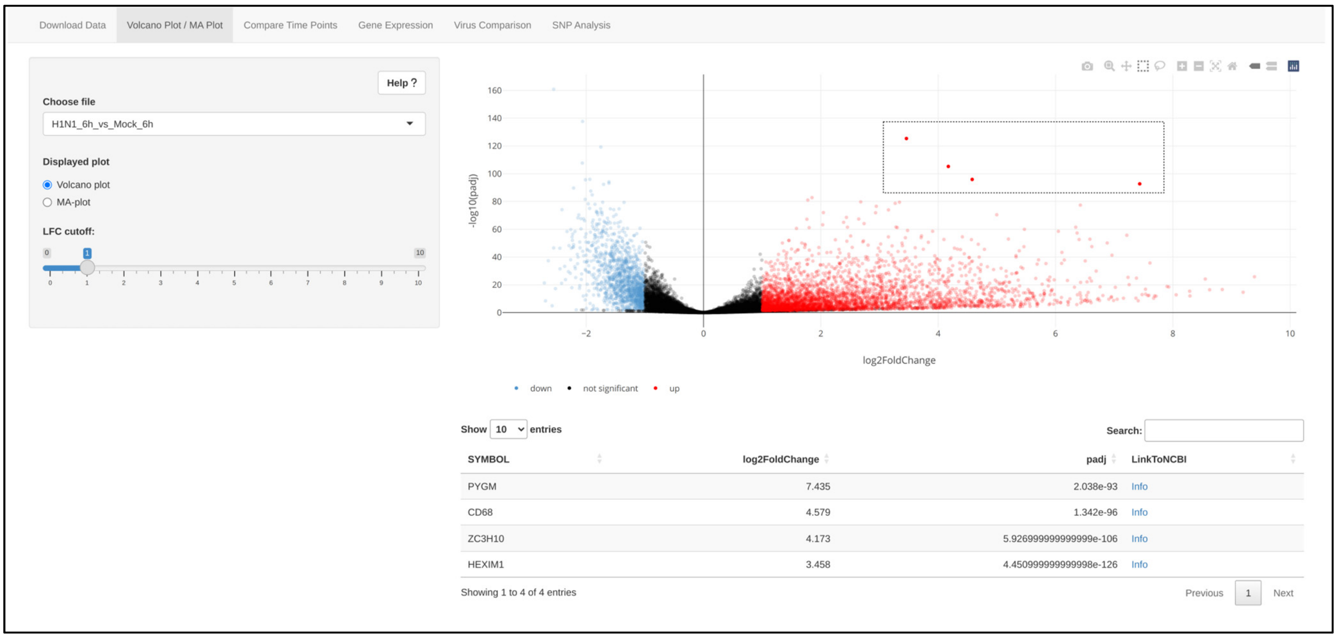

B

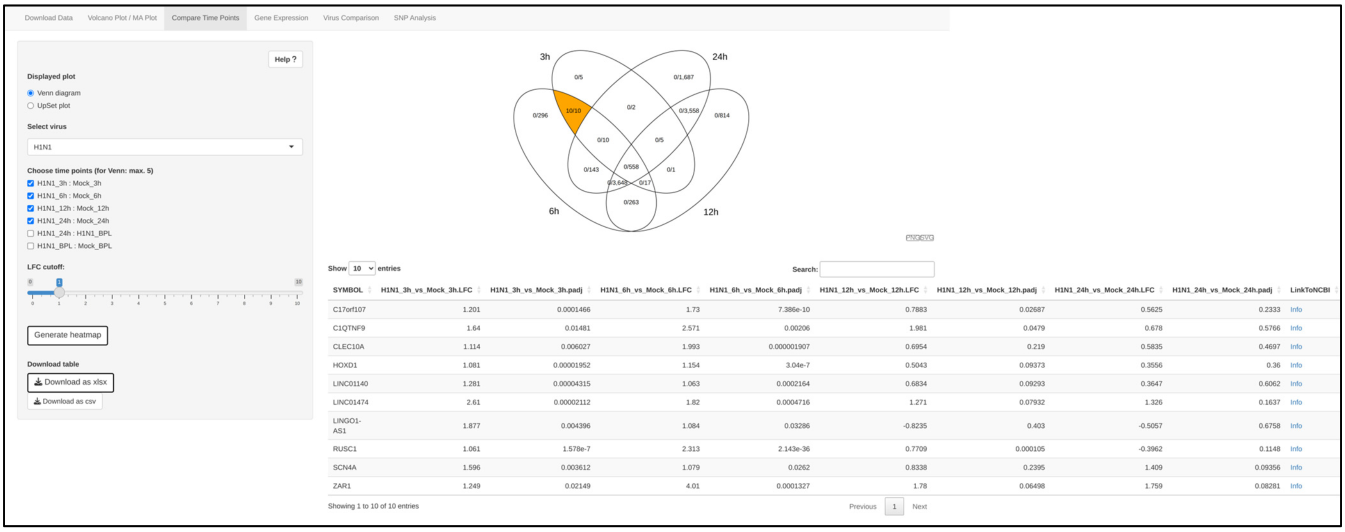

Suppl. Fig. S3. Overview of the different analysis tabs in the ADVICER application illustrated by examples. (A) The tab 'Volcano plot / MA plot' shows a Volcano plot for the selected sample (e.g., H1N1 6 h post infection versus mock control.) with DEGs colored in red (up-regulated) or blue (down-regulated). Subsets of genes can be highlighted and listed in a table. **(B)** The tab 'Compare Time Points' shows a Venn diagram for the selected virus and time points. The genes in the chosen intersection (orange) are listed as a table containing the gene symbol, log2 fold change (LFC), and adjusted *P* value (padj) for all conditions and a link to NCBI.

C

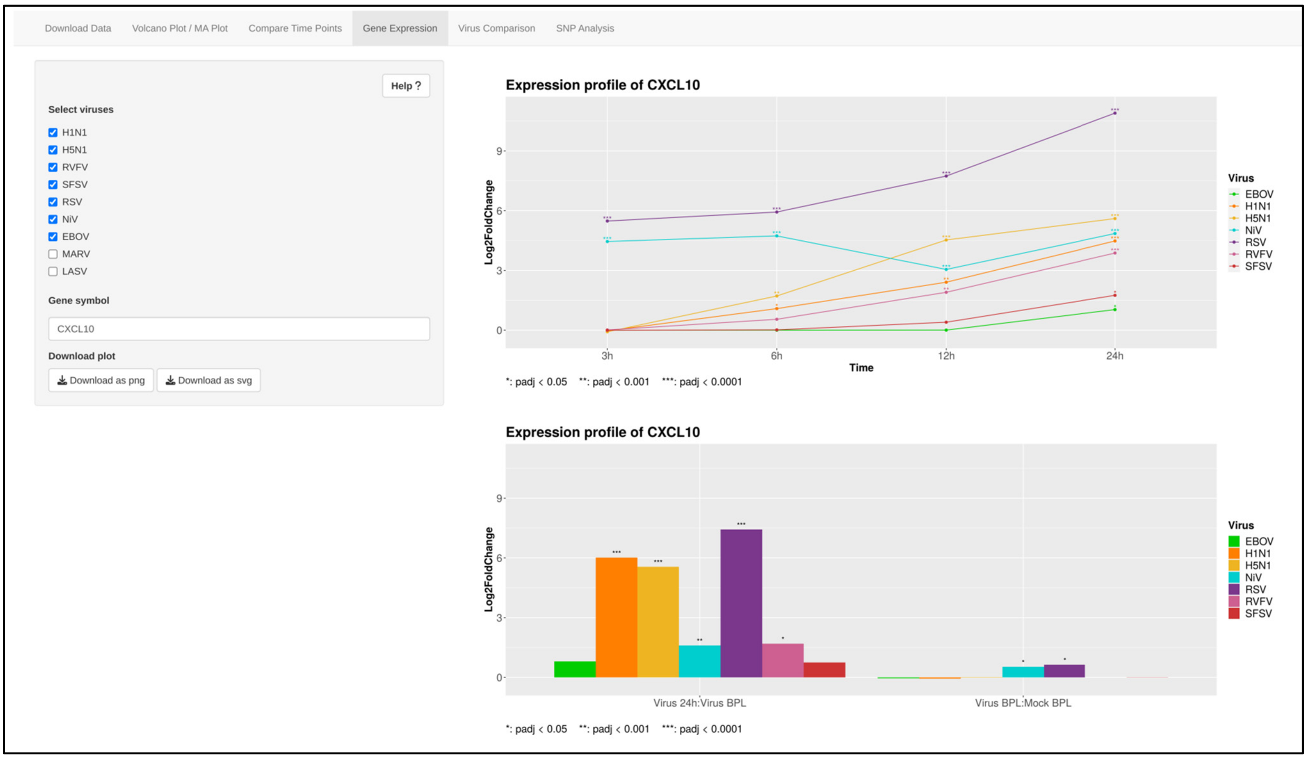

D

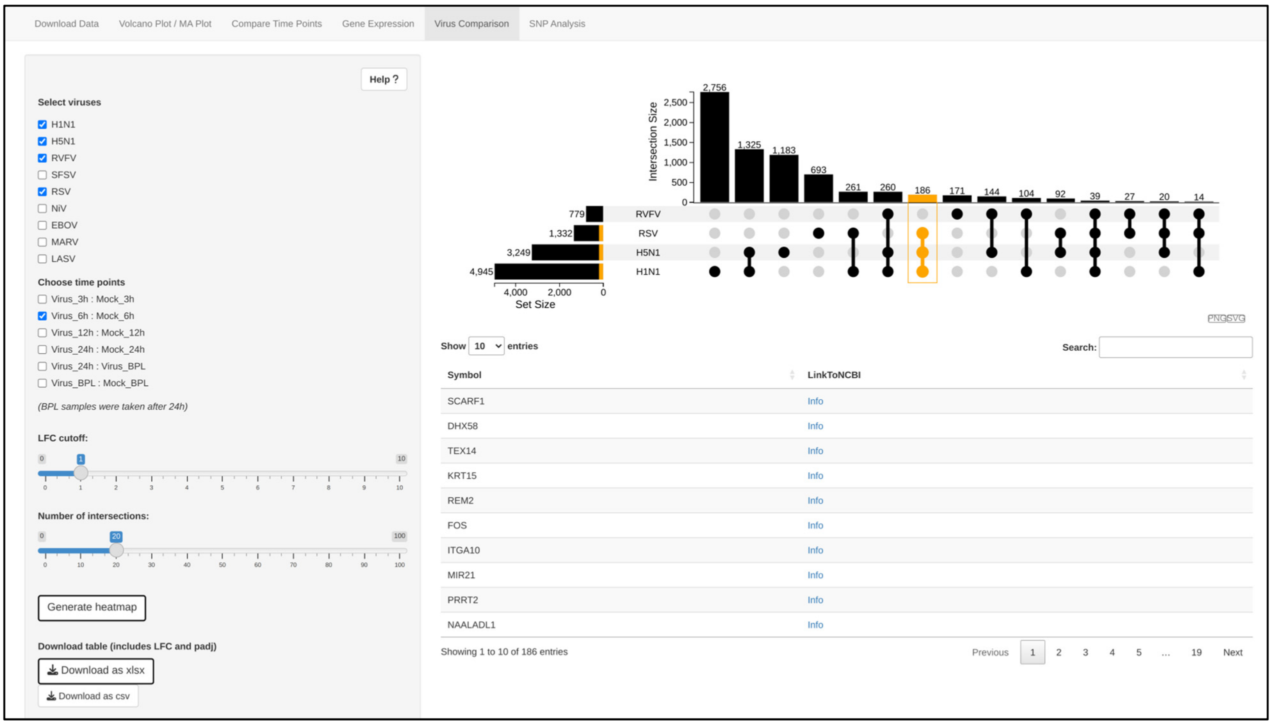

Suppl. Fig. S3. Continued from previous page. **(C)** The tab 'Gene Expression' plots the LFC of a specific gene for all chosen viruses over time with asterisks representing the significance of the fold change. The different viruses are indicated by different colors. **(D)** The tab 'Virus Comparison' shows an UpSet plot comparing the genes differentially expressed in at least one of the selected time points for the chosen viruses. Genes of the selected intersection (orange) are displayed as a table containing the gene symbol and a link to NCBI.

E

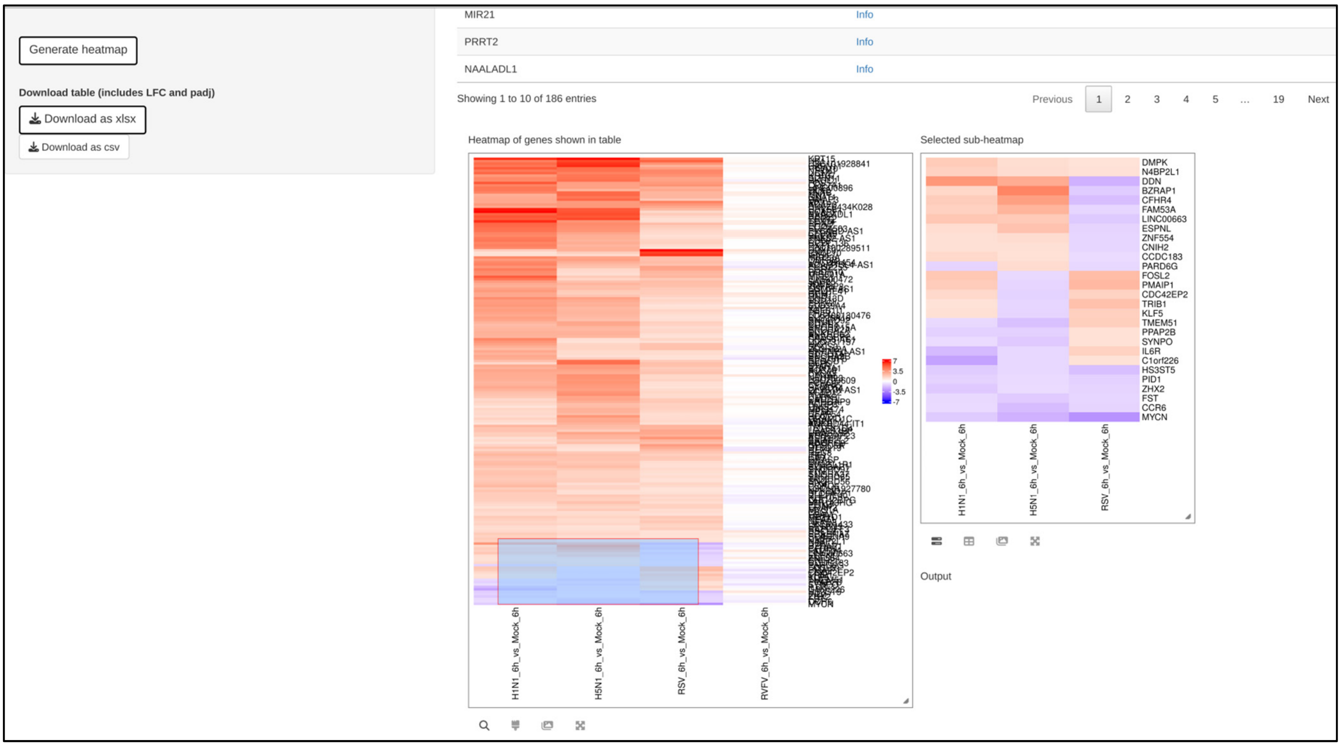

Suppl. Fig. S3. Continued from previous page. The chosen intersection in (B) and (D) can also be plotted as a heat map depicting the LFC for all included data **(E)**.

	H1N1	H5N1	RVFV	SFSV	RSV	NiV	EBOV	MARV	LASV
**Producer cell line**	MDCKII	MDCKII	VeroB4	VeroB4	Hep2	Vero76	VeroE6	VeroE6	VeroE6
**Purification of stock virus**	Amicon	Amicon	Amicon	Amicon	UC	UC	UC	UC	UC

HuH7
24 hpi

HuH7
Mock

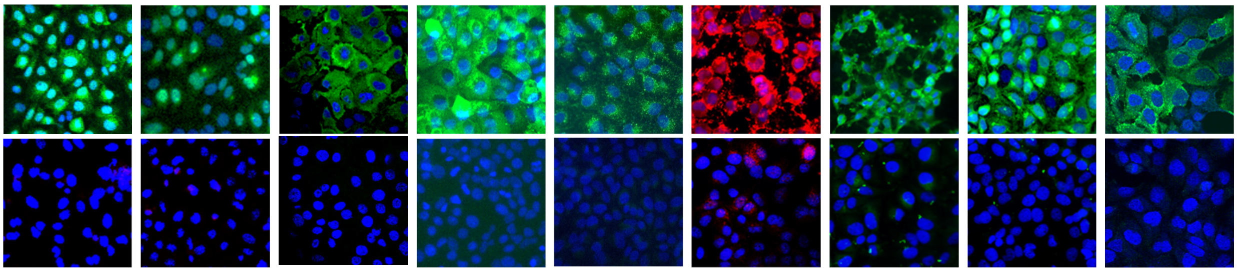

Suppl. Fig. S4. Description of stock virus production. Upper: All viruses were grown in their standard producer cell lines and supernatants were purified and concentrated by Amicon filtration or ultracentrifugation (UC) as shown. Lower: In parallel with the transcriptome study, HuH7 cells grown on coverslips were infected with the viruses shown at the top and fixed at 24 hpi. After fixation and inactivation, infected cells were stained with corresponding virus-specific antibodies and fluorescently-labelled secondary antibodies. Cell nuclei were stained with DAPI (4′,6-diamidino-2-phenylindole).
