# Supplementary material for: Distinct negative-sense RNA viruses induce a common set of transcripts encoding proteins forming an extensive network
Source: J Virol. 2024 Sep 16;98(10):e00935-24. doi: 10.1128/jvi.00935-24 (PMC11494938; doi:10.1128/jvi.00935-24)
Supplement: Table S2 — 47 genes regulated by all viruses with a known function in infection by RNA or DNA viruses. [file jvi.00935-24-s0003.docx]

**Suppl. Table S2.** The table represents 47 genes regulated by all viruses with a known function in infection by RNA or DNA viruses, representative key references describing their function are provided.

| **Gene** | **Reference** | **Gene** | **Reference** | **Gene** | **Reference** |
| --- | --- | --- | --- | --- | --- |
| AFF4 | (1) | EREG | (2) | PLA2G4C | (3) |
| AREG | (4) | FOS | (5) | PLK2 | (6) |
| ARG1 | (7) | FOSL1 | (8) | PPP1R15A | (9) |
| ATF3 | (10) | GADD45B | (11) | RELB | (12) |
| BBC3 | (13) | GEM | (14) | RND1 | (15) |
| BIK | (16) | HDAC9 | (17) | SERTAD1 | (18) |
| BIRC3 | (19) | ID3 | (20) | SLC19A1 | (21) |
| CCDC85B | (22) | KLF4 | (23) | SOCS2 | (24) |
| CCR6 | (25) | KLRC2 | (26) | TAP1 | (27) |
| CREBRF | (28) | LIG4 | (29) | TGFB2 | (30) |
| CXCL1 | (31) | LMNB1 | (32) | TNFAIP3 | (33) |
| CXCL3 | (34) | MCOLN3 | (35) | UHRF1 | (36) |
| CYLD | (37) | MSH2 | (38) | ZC3H12A | (39) |
| DUSP1 | (40) | MXD1 | (41) | ZEB2 | (42) |
| EGR1 | (43) | NEURL3 | (44) | ZFP36 | (45) |
| ELF5 | (46) | NFAT5 | (47) |  |  |

**References**

1. He N, Liu M, Hsu J, Xue Y, Chou S, Burlingame A, Krogan NJ, Alber T, Zhou Q. 2010. HIV-1 Tat and host AFF4 recruit two transcription elongation factors into a bifunctional complex for coordinated activation of HIV-1 transcription. Mol Cell 38:428-38.

2. Ding X, Wang F, Duan M, Yang J, Wang S. 2009. Epiregulin as a key molecule to suppress hepatitis B virus propagation in vitro. Arch Virol 154:9-17.

3. Mishchenko EL, Makarova AA, Antropova EA, Venzel AS, Ivanisenko TV, Demenkov PS, Ivanisenko VA. 2023. Molecular-genetic pathways of hepatitis C virus regulation of the expression of cellular factors PREB and PLA2G4C, which play an important role in virus replication. Vavilovskii Zhurnal Genet Selektsii 27:776-783.

4. Pei R, Chen H, Lu L, Zhu W, Beckebaum S, Cicinnati V, Lu M, Chen X. 2011. Hepatitis C virus infection induces the expression of amphiregulin, a factor related to the activation of cellular survival pathways and required for efficient viral assembly. J Gen Virol 92:2237-2248.

5. Li X, Feng J, Chen S, Peng L, He WW, Qi J, Deng H, Sun R. 2010. Tpl2/AP-1 enhances murine gammaherpesvirus 68 lytic replication. J Virol 84:1881-90.

6. Quan R, Wei L, Hou L, Wang J, Zhu S, Li Z, Lv M, Liu J. 2020. Proteome Analysis in a Mammalian Cell line Reveals that PLK2 is Involved in Avian Metapneumovirus Type C (aMPV/C)-Induced Apoptosis. Viruses 12.

7. Clement M, Ladell K, Miners KL, Marsden M, Chapman L, Cardus Figueras A, Scott J, Andrews R, Clare S, Kriukova VV, Lupyr KR, Britanova OV, Withers DR, Jones SA, Chudakov DM, Price DA, Humphreys IR. 2023. Inhibitory IL-10-producing CD4(+) T cells are T-bet-dependent and facilitate cytomegalovirus persistence via coexpression of arginase-1. Elife 12.

8. Cai B, Wu J, Yu X, Su XZ, Wang RF. 2017. FOSL1 Inhibits Type I Interferon Responses to Malaria and Viral Infections by Blocking TBK1 and TRAF3/TRIF Interactions. mBio 8.

9. Zhu T, Jiang X, Xin H, Zheng X, Xue X, Chen JL, Qi B. 2021. GADD34-mediated dephosphorylation of eIF2alpha facilitates pseudorabies virus replication by maintaining de novo protein synthesis. Vet Res 52:148.

10. Sood V, Sharma KB, Gupta V, Saha D, Dhapola P, Sharma M, Sen U, Kitajima S, Chowdhury S, Kalia M, Vrati S. 2017. ATF3 negatively regulates cellular antiviral signaling and autophagy in the absence of type I interferons. Sci Rep 7:8789.

11. Hu HL, Srinivas KP, Wang S, Chao MV, Lionnet T, Mohr I, Wilson AC, Depledge DP, Huang TT. 2022. Single-cell transcriptomics identifies Gadd45b as a regulator of herpesvirus-reactivating neurons. EMBO Rep 23:e53543.

12. Zhang J, Wang C, Tuo X, Chai K, Xu Y, Qiao W, Tan J. 2020. Prototype foamy virus downregulates RelB expression to facilitate viral replication. FEBS Open Bio 10:2137-2148.

13. Wyler E, Menegatti J, Franke V, Kocks C, Boltengagen A, Hennig T, Theil K, Rutkowski A, Ferrai C, Baer L, Kermas L, Friedel C, Rajewsky N, Akalin A, Dolken L, Grasser F, Landthaler M. 2017. Widespread activation of antisense transcription of the host genome during herpes simplex virus 1 infection. Genome Biol 18:209.

14. Chevalier SA, Turpin J, Cachat A, Afonso PV, Gessain A, Brady JN, Pise-Masison CA, Mahieux R. 2014. Gem-induced cytoskeleton remodeling increases cellular migration of HTLV-1-infected cells, formation of infected-to-target T-cell conjugates and viral transmission. PLoS Pathog 10:e1003917.

15. Kumar A, Mishra S, Kumar A, Raut AA, Sato S, Takaoka A, Kumar H. 2022. Essential role of Rnd1 in innate immunity during viral and bacterial infections. Cell Death Dis 13:520.

16. Mebratu YA, Tipper J, Chand HS, Walton S, Harrod KS, Tesfaigzi Y. 2016. Bik Mediates Caspase-Dependent Cleavage of Viral Proteins to Promote Influenza A Virus Infection. Am J Respir Cell Mol Biol 54:664-73.

17. Hou S, Wang X, Ren S, Meng X, Yin X, Zhang J, Tarasiuk K, Pejsak Z, Jiang T, Mao R, Zhang Y, Sun Y. 2022. Knockout of HDAC9 Gene Enhances Foot-and-Mouth Disease Virus Replication. Front Microbiol 13:805606.

18. Vuono EA, Ramirez-Medina E, Azzinaro P, Berggren KA, Rai A, Pruitt S, Silva E, Velazquez-Salinas L, Borca MV, Gladue DP. 2020. SERTA Domain Containing Protein 1 (SERTAD1) Interacts with Classical Swine Fever Virus Structural Glycoprotein E2, Which Is Involved in Virus Virulence in Swine. Viruses 12.

19. Ebert G, Preston S, Allison C, Cooney J, Toe JG, Stutz MD, Ojaimi S, Scott HW, Baschuk N, Nachbur U, Torresi J, Chin R, Colledge D, Li X, Warner N, Revill P, Bowden S, Silke J, Begley CG, Pellegrini M. 2015. Cellular inhibitor of apoptosis proteins prevent clearance of hepatitis B virus. Proc Natl Acad Sci U S A 112:5797-802.

20. Menner AJ, Rauch KS, Aichele P, Pircher H, Schachtrup C, Schachtrup K. 2015. Id3 Controls Cell Death of 2B4+ Virus-Specific CD8+ T Cells in Chronic Viral Infection. J Immunol 195:2103-14.

21. Miyake A, Kawasaki J, Ngo H, Makundi I, Muto Y, Khan AH, Smith DJ, Nishigaki K. 2019. Reduced Folate Carrier: an Entry Receptor for a Novel Feline Leukemia Virus Variant. J Virol 93.

22. Brazas R, Ganem D. 1996. A cellular homolog of hepatitis delta antigen: implications for viral replication and evolution. Science 274:90-4.

23. Luo WW, Lian H, Zhong B, Shu HB, Li S. 2016. Krüppel-like factor 4 negatively regulates cellular antiviral immune response. Cell Mol Immunol 13:65-72.

24. Toscano ECB, Sousa L, Lima GK, Mesquita LA, Vilela MC, Rodrigues DH, Ferreira RN, Soriani FM, Campos MA, Kroon EG, Teixeira MM, de Miranda AS, Rachid MA, Teixeira AL. 2020. Neuroinflammation is associated with reduced SOCS2 and SOCS3 expression during intracranial HSV-1 infection. Neurosci Lett 736:135295.

25. Lee AYS, Korner H. 2017. CCR6/CCL20 chemokine axis in human immunodeficiency virus immunity and pathogenesis. J Gen Virol 98:338-344.

26. Vietzen H, Zoufaly A, Traugott M, Aberle J, Aberle SW, Puchhammer-Stockl E. 2021. Deletion of the NKG2C receptor encoding KLRC2 gene and HLA-E variants are risk factors for severe COVID-19. Genet Med 23:963-967.

27. Xia Z, Xu G, Yang X, Peng N, Zuo Q, Zhu S, Hao H, Liu S, Zhu Y. 2017. Inducible TAP1 Negatively Regulates the Antiviral Innate Immune Response by Targeting the TAK1 Complex. J Immunol 198:3690-3704.

28. Audas TE, Hardy-Smith PW, Penney J, Taylor T, Lu R. 2016. Characterization of nuclear foci-targeting of Luman/CREB3 recruitment factor (LRF/CREBRF) and its potential role in inhibition of herpes simplex virus-1 replication. Eur J Cell Biol 95:611-622.

29. Muylaert I, Elias P. 2007. Knockdown of DNA ligase IV/XRCC4 by RNA interference inhibits herpes simplex virus type I DNA replication. J Biol Chem 282:10865-72.

30. Thomas BJ, Kan OK, Loveland KL, Elias JA, Bardin PG. 2016. In the Shadow of Fibrosis: Innate Immune Suppression Mediated by Transforming Growth Factor-beta. Am J Respir Cell Mol Biol 55:759-766.

31. Korbecki J, Maruszewska A, Bosiacki M, Chlubek D, Baranowska-Bosiacka I. 2022. The Potential Importance of CXCL1 in the Physiological State and in Noncancer Diseases of the Cardiovascular System, Respiratory System and Skin. Int J Mol Sci 24.

32. Mou F, Wills EG, Park R, Baines JD. 2008. Effects of lamin A/C, lamin B1, and viral US3 kinase activity on viral infectivity, virion egress, and the targeting of herpes simplex virus U(L)34-encoded protein to the inner nuclear membrane. J Virol 82:8094-104.

33. Maelfait J, Roose K, Bogaert P, Sze M, Saelens X, Pasparakis M, Carpentier I, van Loo G, Beyaert R. 2012. A20 (Tnfaip3) deficiency in myeloid cells protects against influenza A virus infection. PLoS Pathog 8:e1002570.

34. Hickman HD, Reynoso GV, Ngudiankama BF, Cush SS, Gibbs J, Bennink JR, Yewdell JW. 2015. CXCR3 chemokine receptor enables local CD8(+) T cell migration for the destruction of virus-infected cells. Immunity 42:524-37.

35. Santoni G, Morelli MB, Amantini C, Nabissi M, Santoni M, Santoni A. 2020. Involvement of the TRPML Mucolipin Channels in Viral Infections and Anti-viral Innate Immune Responses. Front Immunol 11:739.

36. Wang M, Song J, Gao C, Yu C, Qin C, Lang Y, Xu A, Liu Y, Feng W, Tang J, Zhang R. 2023. UHRF1 Deficiency Inhibits Alphaherpesvirus through Inducing RIG-I-IRF3-Mediated Interferon Production. J Virol 97:e0013423.

37. Zhang L, Wei N, Cui Y, Hong Z, Liu X, Wang Q, Li S, Liu H, Yu H, Cai Y, Wang Q, Zhu J, Meng W, Chen Z, Wang C. 2018. The deubiquitinase CYLD is a specific checkpoint of the STING antiviral signaling pathway. PLoS Pathog 14:e1007435.

38. Jenab-Wolcott J, Rodriguez-Correa D, Reitmair AH, Mak T, Rosenberg N. 2000. The absence of Msh2 alters abelson virus pre-B-cell transformation by influencing p53 mutation. Mol Cell Biol 20:8373-81.

39. Li Y, Que L, Fukano K, Koura M, Kitamura K, Zheng X, Kato T, Aly HH, Watashi K, Tsukuda S, Aizaki H, Watanabe N, Sato Y, Suzuki T, Suzuki HI, Hosomichi K, Kurachi M, Wakae K, Muramatsu M. 2020. MCPIP1 reduces HBV-RNA by targeting its epsilon structure. Sci Rep 10:20763.

40. Caceres A, Perdiguero B, Gomez CE, Cepeda MV, Caelles C, Sorzano CO, Esteban M. 2013. Involvement of the cellular phosphatase DUSP1 in vaccinia virus infection. PLoS Pathog 9:e1003719.

41. Sjöblom-Hallén A, Yang W, Jansson A, Rymo L. 1999. Silencing of the Epstein-Barr virus latent membrane protein 1 gene by the Max-Mad1-mSin3A modulator of chromatin structure. J Virol 73:2983-93.

42. Minor MM, Hollinger FB, McNees AL, Jung SY, Jain A, Hyser JM, Bissig KD, Slagle BL. 2020. Hepatitis B Virus HBx Protein Mediates the Degradation of Host Restriction Factors through the Cullin 4 DDB1 E3 Ubiquitin Ligase Complex. Cells 9.

43. Woodson CM, Kehn-Hall K. 2022. Examining the role of EGR1 during viral infections. Front Microbiol 13:1020220.

44. Zhao Y, Cao X, Guo M, Wang X, Yu T, Ye L, Han L, Hei L, Tao W, Tong Y, Xu Y, Zhong J. 2018. Neuralized E3 Ubiquitin Protein Ligase 3 Is an Inducible Antiviral Effector That Inhibits Hepatitis C Virus Assembly by Targeting Viral E1 Glycoprotein. J Virol 92.

45. Maeda M, Sawa H, Tobiume M, Tokunaga K, Hasegawa H, Ichinohe T, Sata T, Moriyama M, Hall WW, Kurata T, Takahashi H. 2006. Tristetraprolin inhibits HIV-1 production by binding to genomic RNA. Microbes Infect 8:2647-56.

46. Pietzner M, Chua RL, Wheeler E, Jechow K, Willett JDS, Radbruch H, Trump S, Heidecker B, Zeberg H, Heppner FL, Eils R, Mall MA, Richards JB, Sander LE, Lehmann I, Lukassen S, Wareham NJ, Conrad C, Langenberg C. 2022. ELF5 is a potential respiratory epithelial cell-specific risk gene for severe COVID-19. Nat Commun 13:4484.

47. Ranjbar S, Tsytsykova AV, Lee SK, Rajsbaum R, Falvo JV, Lieberman J, Shankar P, Goldfeld AE. 2006. NFAT5 regulates HIV-1 in primary monocytes via a highly conserved long terminal repeat site. PLoS Pathog 2:e130.
